# Sex-Specific Differences of Adenosine Triphosphate Levels in Red Blood Cells Isolated From ApoE/LDLR Double-Deficient Mice

**DOI:** 10.3389/fphys.2022.839323

**Published:** 2022-02-18

**Authors:** Fatih Celal Alcicek, Tasnim Mohaissen, Katarzyna Bulat, Jakub Dybas, Ewa Szczesny-Malysiak, Magdalena Kaczmarska, Magdalena Franczyk-Zarow, Renata Kostogrys, Katarzyna M. Marzec

**Affiliations:** ^1^Jagiellonian Centre for Experimental Therapeutics, Jagiellonian University, Krakow, Poland; ^2^Chair and Faculty of Pharmacy, Jagiellonian University Medical College, Krakow, Poland; ^3^Łukasiewicz Research Network - Krakow Institute of Technology, Krakow, Poland; ^4^Department of Human Nutrition and Dietetics, Faculty of Food Technology, University of Agriculture, Krakow, Poland

**Keywords:** ApoE/LDLR^−/−^ mice, intracellular ATP, atherosclerosis, red blood cells, sex-related differences, extracellular ATP

## Abstract

In this study for the first time, we investigated the correlation between sex-specific differences in adenosine triphosphate (ATP) levels in red blood cells (RBCs) and their mechanical, biochemical, and morphological alterations during the progression of atherosclerosis in ApoE/LDLR double-deficient (ApoE/LDLR^−/−^) mice. Our results indicate that both sex and age affect alterations in RBCs of both ApoE/LDLR^−/−^ and C57BL/6J mice. When compared with male RBCs, female RBCs were characterized by lower basal ATP and mean corpuscular hemoglobin concentration (MCHC), higher hemoglobin concentration (HGB), mean corpuscular volume (MCV), mean corpuscular hemoglobin (MCH), deformability, and phosphatidylserine (PS) exposure levels, regardless of age in both, ApoE/LDLR^−/−^ and C57BL/6J mice. ApoE/LDLR^−/−^ mice compared with age-matched controls showed lower basal ATP levels regardless of age and sex. Intracellular ATP level of RBCs was decreased solely in senescent female C57BL/6J mice, while it was elevated in males. Basal extracellular ATP levels were 400 times lower than corresponding intracellular level. In conclusion, basal ATP levels, RBC morphology, deformability, PS exposure levels alterations are sex-dependent in mice. Changes in basal ATP levels were correlated with PS exposure and trends of changes in MCV. Trends of changes of the most RBC parameters were similar in both sexes of ApoE/LDLR^−/−^ mice compared with age-matched controls; however, their kinetics and levels vary greatly between different stages of disease progression.

## Introduction

Atherosclerosis, one of the major causes of mortality worldwide, is a disease that involves endothelial dysfunction and lipid retention in vascular intima, as the result of hyperlipidemia and lipid oxidation. Pathogenesis and pharmacotherapy of atherosclerosis are well established, though mainly focused on the vascular endothelium (Bergheanu et al., 2017). Although it has been shown that red blood cells (RBCs) are involved in the plaque expansion in the vascular endothelium (Jeney et al., 2014), further investigations on the role of RBC alterations in the development of atherosclerosis could pave the way to better understanding and possible treatment opportunities of the disease in men and women. Therefore, it would be beneficial to reveal physiological alterations in female and male RBCs during progression of atherosclerosis.

Red blood cells transport oxygen from the respiratory epithelium to tissues and cells. They are disk-shaped cells with a flatter, concave center, and their size and lifespan vary among species. Murine RBCs have a thickness of 2 μm, a diameter of 4–7 μm, and approximately 45 days of average lifespan (Gottlieb et al., 2012; O’Connell et al., 2015). Physiological properties and sex-related differences of RBCs, as well as their role in pathophysiological processes, were documented and widely reviewed (Murphy, 2014; Said et al., 2015; Kuhn et al., 2017; Grau et al., 2018; Huisjes et al., 2018; Pretini et al., 2019; Mohaissen et al., 2021; Dybas et al., 2022). Deformability is the ability of RBCs to change their shape during passing through the tiniest capillaries and is a crucial property for sufficient oxygenation of tissues (Huisjes et al., 2018). RBC deformability was found to be sex-related and impaired in various diseases (Kameneva et al., 1999, 2003; Nemeth et al., 2010; Grau et al., 2018; Somogyi et al., 2018). Some of the morphological parameters of RBCs, such as hemoglobin concentration (HGB), mean corpuscular volume (MCV), mean corpuscular hemoglobin (MCH), and mean corpuscular hemoglobin concentration (MCHC), differ between males and females, and were also previously reported to be altered to different extent in pathophysiological processes, in a sex-dependent manner (Straface et al., 2011; Kanias et al., 2016; Grau et al., 2018; Szczesny-Malysiak et al., 2021). It was also previously reported that progression of atherosclerosis induces characteristic mechanical, morphological, and physiological RBC alterations (Kanakaraj and Singh, 1989; Van Zwieten et al., 2012; Unruh et al., 2015; Dybas et al., 2020), but yet, the impact of sex has not been investigated in ApoE-LDLR double-deficient (ApoE/LDLR^−/−^) mice.

One of the important physiological parameters is the level of intracellular adenosine triphosphate (ATP) molecules, which is essential for RBCs to maintain their viability. ATP is known as the energy source for cells and its depletion was previously reported in advanced atherosclerosis (Ekstrand et al., 2017). A decline in intracellular ATP level in RBCs below a certain level causes activation of apoptosis (de Korte et al., 1985). Besides maintaining viability, ATP also regulates various physiological aspects, such as cell shape, deformability, and phospholipid asymmetry of RBC membrane (especially phosphatidylserine (PS) exposure, which is a marker of eryptosis or RBCs damage) (Feo and Mohandas, 1977; Seigneuret and Devaux, 1984; Betz et al., 2009; Xu et al., 2019). Moreover, RBCs release ATP spontaneously with a constant rate when no perturbation occurs, constituting a steady extracellular ATP pool defined here as “basal extracellular ATP level” (Montalbetti et al., 2011; Leal Denis et al., 2013). ATP release from RBCs can be modified in response to various physiological and pharmacological stimuli what became subject of this work. RBC-derived ATP has an important role in microcirculation, that is, to regulate the blood flow and consequently the oxygenation of tissues (Sprague et al., 2007).

In this work, we characterized sex-related alterations in RBCs in correlation to their ATP levels in ApoE/LDLR^−/−^ mice. These mice were found to be a reliable model of human atherosclerosis, which shows impaired lipoprotein clearance (Sasso et al., 2016). Consequently, hypercholesterolemia develops from an early age and atherosclerotic lesions begin to occur between 12 and 16 weeks of age, even when fed a normal diet (Wojewoda et al., 2016; Bar et al., 2019). Therefore, in mice of chosen ages (8 and 24 weeks old), we observed the effect of hypercholesterolemia and developed atherosclerotic disease, respectively. In this paper, we present a comprehensive analysis of ATP profile in female and male RBCs in relation to their morphology, deformability, and PS exposure levels on the membrane surface. The analysis of data in relation to hypercholesterolemia and developed atherosclerosis was supported by analysis of biochemistry of blood plasma, including plasma lipid profile. To the best of our knowledge, this is the first time when sex-related differences in ATP profile of RBCs isolated from ApoE/LDLR^−/−^ mice compared to control mice were evaluated.

## Materials and Methods

### Animal Models

Murine model of atherosclerosis (8 and 24 weeks old, female and male, *N* = 6–8), ApoE/LDLR^−/−^ (Ishibashi et al., 1994), bred at the University of Agriculture in Krakow, and wild-type controls (8- and 24-week-old, female and male, *N* = 6–9), C57BL/6J, bred at the Jackson Lab (Bar Harbor, Maine, United States), were used for the experiments. Mice were housed at the animal facility of Jagiellonian Centre for Experimental Therapeutics, Jagiellonian University in Krakow, in the conditions of 12-h light/dark cycle, standard rodent chow diet, and unlimited access to drinking water. All animal procedures were performed in accordance with the Guide for the Care and Use of Laboratory Animals published by the United States National Institutes of Health (NIH Publication No. 85–23, revised 1985) as well as with the guidelines of the Local Ethical Committee on Animal Experiments in Krakow. The number of mice in each given experimental group is stated in the caption of the corresponding figure.

### Blood Collection and RBC Isolation

Before sacrifice, mice were anesthetized *via* intraperitoneal injection of overdose of ketamine (100 mg/kg) and xylazine (10 mg/kg) mixture. Whole blood samples were collected directly from the right ventricle using 21G needle and gently aspiration to a 2 ml syringe containing heparin as an anticoagulant (10 units/ml). Immediately after blood collection, the complete blood count was performed. Whole blood samples were subjected to centrifugation (acceleration: 500 × *g*, run time: 10 min, 4°C, soft stop) up to 1 h after collection. The plasma and buffy coat were aspirated after centrifugation. RBCs were washed with the daily buffer solution (DBS). Subsequently, centrifugation was repeated two times followed by removal of supernatant and buffy coat. Each time, DBS was added to triple the total volume of the remained sample to rinse RBCs. The DBS was prepared on the same day for each experiment, and it contained: 21 mM Tris Base, 140.5 mM NaCl, 4.7 mM KCl, 2 mM CaCl_2_, 1.2 mM MgSO_4_, 5.5 mM glucose, and 76 μM bovine serum albumin, with a final pH adjusted to 7.40. All chemicals were dissolved in distilled water and the solution was filtered through a pleated filter with 0.22 μm pore size.

### RBC Morphology and Deformability Measurements

After collection, all whole blood samples were subjected to complete blood count analysis (RBC, HCT, HGB, MCV, MCH, and MCHC) using the AbcVet hematology analyzer (Horiba Medical, Montpellier, France).

All measurements of RBC deformability were performed on a RheoScan AnD 300 (RheoMeditech, Seoul, Korea) slit–flow ektacytometer (Shin et al., 2005; Baskurt et al., 2009a,b). For determination of elongation index (EI), 6 μl of blood was suspended in 600 μl isotonic viscous polymer solution [polyvinylpyrrolidone (PVP), 360 kDa, RheoMeditech, Seoul, South Korea]. The physicochemical properties of this buffer were as follows: pH of 7.4, osmotic pressure of 310 mOsm/kg, and viscosity of 30 ± 2 mPa·s. Prior to measurement, the solution was heated to 37°C and total volume of 500 μl of the blood–PVP solution was transferred to the plastic disposable reservoir provided by the manufacturer. Subsequently, fitted results were displayed as a relationship between EI and applied shear stress (ranging between 0.5 and 20 Pa). The EI value was calculated using the formula EI = (L − W)/(L + W), where L is the length and W is the width of the diffraction pattern of RBCs flowing through the reservoir’s microchannel formed when 633–nm laser light was applied. Erythrocyte deformability results are shown as EI measured at the highest shear stresses (20 Pa).

### Assessment of Phosphatidylserine Expression

Volume of 1 μl of isolated red blood cells was diluted in 100 μl of Annexin Binding Buffer diluted 1:10 with 0.9% saline containing 0.05% of nuclei stain-Hoechst 33342 (Thermo Scientific cat. No. H3570), rat anti-mouse anti-TER-119-PerCP Cy5.5 (erythroid cell marker, 1:200, BioLegend cat. No. 116228) and Annexin V-FITC (a marker of apoptotic cells, 1:200, BD cat. No. 556547). The cells were incubated in the dark (30 min, RT) and diluted in 400 μl of PBS. Cellular analysis was performed with LSRII flow cytometer (BD Biosciences, Franklin Lakes, United States), 1 million events were recorded for each sample. The data were analyzed with use of FASC DIVA software.

### Biochemical Analysis of Blood Plasma

The plasma withdrawn after the first centrifugation of the blood samples was used for the biochemical measurements using a colorimetric-based biochemical analyzer ABX Pentra 400 (Horiba Medical, Kyoto, Japan). The levels of cholesterol, high density lipoprotein (HDL), low density lipoprotein (LDL), and triglycerides were assessed using the Horiba ABX reagents and in accordance with the manufacturer’s procedures (including necessary calibrations and controls for analyzed parameters).

### Determination of Intracellular and Extracellular ATP Levels of Murine RBCs

Adenosine triphosphate levels were measured using ATPlite 1step luminescence ATP detection assay system (PerkinElmer, United States), which is based on luciferin-luciferase technique (Strehler, 1968), according to the supplier’s protocol. Light emitted from the reaction of D-luciferin with ATP was measured in each well using a Luminometer LB9508 (Berthold Technologies, Germany). To determine ATP levels, the peak light emitted was compared with an ATP calibration curve generated during the experiment.

Red blood cell suspensions were prepared with 10% hematocrit to determine ATP levels in murine RBCs. Around 10 μl of each sample was taken for intracellular ATP measurement, and 200 μl was taken for the measurement of extracellular ATP level. RBC suspensions were subjected to another complete blood count to define the exact RBC number taken to ATP level calculations.

Volume of 10 μl of each sample were placed in the Eppendorf tubes with 190 μl of deionized water to cause hemolysis (20 times diluted). Subsequently, samples were subjected to sonication for 1 min and to shaking at 700 rpm for 1 min. Two technical replicates, 50 μl of lysate from each sample, were taken for the measurement. Values were normalized to picomoles per 10^6^ RBCs.

Around 200 μl of each sample were placed into the Eppendorf tubes and incubated at 37°C for 10 min. Later, all samples were subjected to centrifugation (acceleration: 1,000 × *g*, run time: 2 min, 4°C, soft stop). Two technical replicates, 50 μl of supernatant from each sample, were taken for the measurement. Values were normalized to picomoles per 4 × 10^8^ RBCs.

### Evaluation of Hemolysis

The presence of Hb in the supernatant was determined with use of a double beam spectrophotometer Lambda 950 (Perkin Elmer, United States) and evaluated to exclude possible significant hemolysis occurred during the incubation of isolated RBC suspensions at 37°C that may affect the determined extracellular ATP levels of RBCs. Absorption spectra were recorded in the 300–700 nm spectral range using a 10 mm path length cuvette. The level of hemolysis was assessed based on the absorbance at 415 nm (Kampen and Zijlstra, 1966). The supernatant from each sample was diluted 50 times with 0.9% NaCl solution prior to measurement.

### Statistical Analysis of Data

All data were analyzed in OriginPro 2018 (OriginLab, United States) software. Statistics were calculated according to the results of normality assessment performed using Shapiro–Wilk test. If the data met normality requirement, significance was checked with one-way ANOVA model with Tukey’s or Holm–Sidak’s *post hoc* test and results were presented as box diagrams with mean, SD, and min–max whiskers. Otherwise, Kruskal–Wallis ANOVA non-parametric test was performed, and results were shown as box diagrams containing mean, median, and interquartile range together with min–max whiskers.

## Results

### Deformability of Murine RBCs

**Figure 1:**
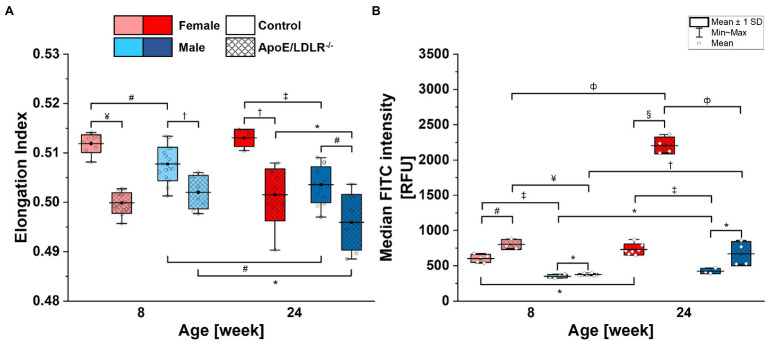
Deformability **(A)** and phosphatidylserine (PS) exposure on the surface **(B)** of red blood cells (RBCs) isolated from 8- and 24-week-old, female and male, control and ApoE/LDLR^−/−^ mice (*N* = 3–9). Normality was assessed with Shapiro–Wilk test, statistical significance was calculated using one-way ANOVA with Tukey’s or Holm–Sidak’s *post hoc* test, and results were presented as box diagrams with mean, SD, and min–max whiskers (^*^p < 0.05, ^#^*p* < 0.01, ^†^*p* < 10^−4^, ^‡^*p* < 10^−5^, ^ф^*p* < 10^−6^, ^¤^*p* < 10^−7^, ^¥^*p* < 10^−8^, and ^§^*p* < 10^−9^).

Red blood cell deformability (Figure 1A) was found to be significantly decreased in ApoE/LDLR^−/−^ mice compared with their age-matched control in both sexes. The deformability of RBCs in female mice was higher than in age-matched male mice in all studied groups, except for 8-week-old ApoE/LDLR^−/−^ mice, which showed no sex-related difference. RBCs isolated from male mice of both control and ApoE/LDLR^−/−^, were characterized by a significant decrease in deformability with age progression, contrary to RBCs acquired from females, where we did not observe any age-related differences.

### Phosphatidylserine Expression on the Membrane of Murine RBCs

Phosphatidylserine exposure on the surface of RBCs (Figure 1B) isolated from ApoE/LDLR^−/−^ mice was significantly elevated compared with their age-matched control in both sexes. RBCs isolated from female mice showed significantly higher PS exposure levels on the surface of their membranes compared with age-matched male mice in both murine models. Moreover, we observed a significant age-dependent increase in PS exposure levels on the surface of RBCs in all studied groups.

### Morphological Parameters of Murine RBCs

**Figure 2:**
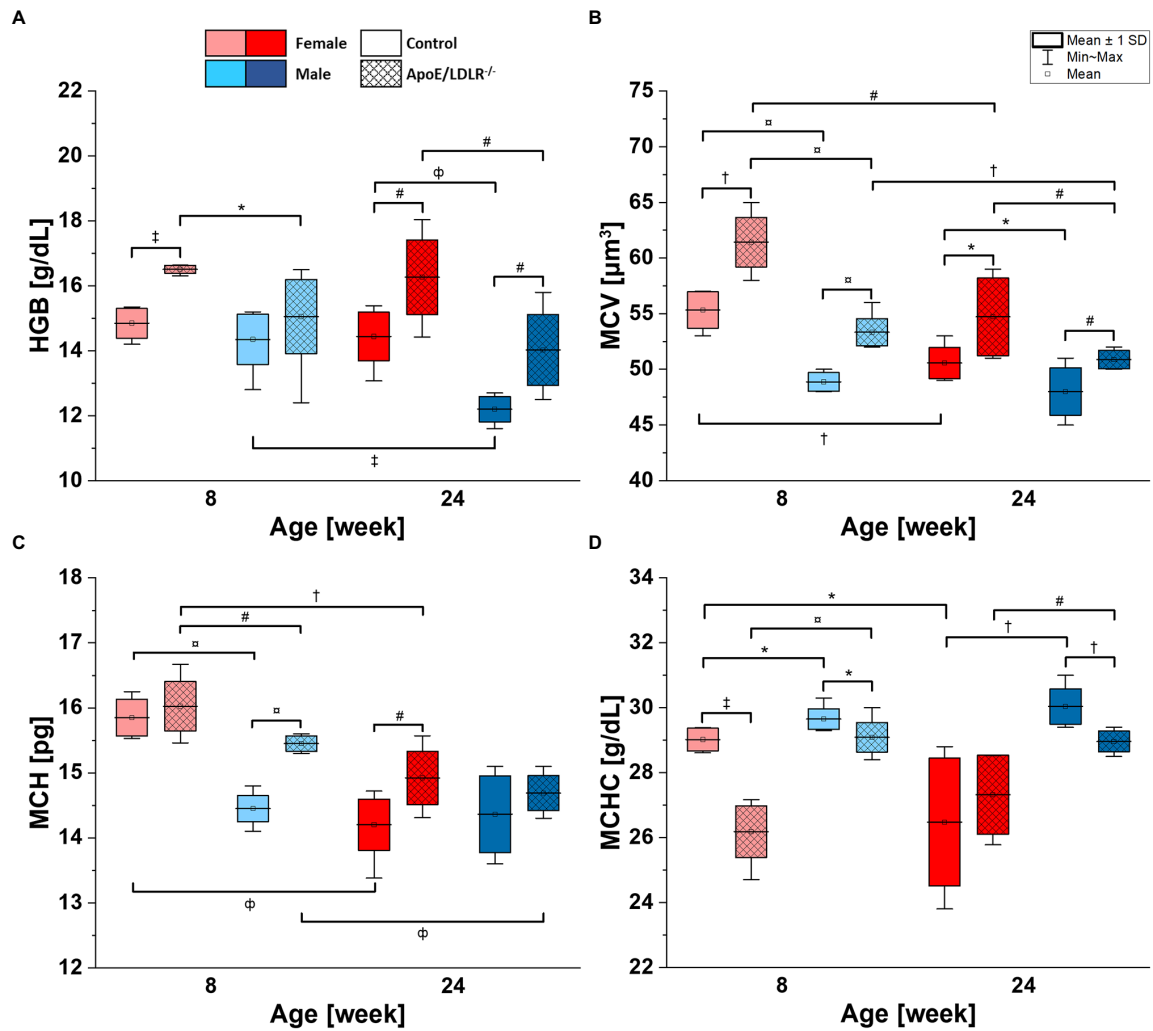
Complete blood count [hemoglobin concentration (HGB; **A**), mean corpuscular volume (MCV; **B**), mean corpuscular hemoglobin (MCH; **C**), and mean corpuscular hemoglobin concentration (MCHC; **D**)] analysis results for 8- and 24-week-old, female and male, control and ApoE/LDLR^−/−^ mice (*N* = 4–9). Normality was assessed using Shapiro–Wilk test, significance was checked with one-way ANOVA model with Tukey’s test, and results were presented as box diagrams with mean, SD, and min–max whiskers (^*^p < 0.05, ^#^*p* < 0.01, ^†^*p* < 10^−4^, ^‡^*p* < 10^−5^, ^ф^*p* < 10^−6^, and ^¤^*p* < 10^−7^).

Lower HGB values were observed in male mice compared with age-matched female mice in both ApoE/LDLR^−/−^ and controls (Figure 2A). The declines were statistically significant in 8- and 24-week-old ApoE/LDLR^−/−^ mice, and in 24-week-old mice control mice. However, we did not observe age-related differences in HGB levels in any studied groups, except for male control mice that showed a decrease in HGB values progressing with age. Moreover, HGB values were higher in RBCs isolated from ApoE/LDLR^−/−^ mice compared with their age-matched controls in all studied groups. Such elevation in HGB values was statistically significant in 8- and 24-week-old female ApoE/LDLR^−/−^ mice, and in 24-week-old male ApoE/LDLR^−/−^ mice.

Mean corpuscular volume values of RBCs (Figure 2B) isolated from ApoE/LDLR^−/−^ mice showed a significant increase in all studied groups compared with age-matched control. Control and ApoE/LDLR^−/−^ male mice showed significantly lower MCV values compared with age-matched female mice. In all studied groups, we observed a significant age-dependent decline in MCV values, except for male control mice.

Higher MCH values in Figure 2C were observed in RBCs isolated from ApoE/LDLR^−/−^ mice compared with their age-matched controls, in both female and male groups. However, only 8-week-old male mice and 24-week-old female mice showed significant elevation. As for the sex-related changes, 8-week-old male control and ApoE/LDLR^−/−^ mice showed a significant decline in MCH values, while no significant difference was observed in 24-week-old male control and ApoE/LDLR^−/−^ mice when compared to age-matched females. Moreover, we observed that MCH values declined significantly with senescence in all studied groups, except for male control mice.

However, we observed significantly lower MCHC values (Figure 2D) in RBCs of ApoE/LDLR^−/−^ mice compared to age-matched control mice in both sex groups, except for 24-week-old females. Male control and ApoE/LDLR^−/−^ mice showed significantly higher MCHC values compared with age-matched females in all studied groups. Yet, we observed no significant age-related differences in MCHC levels in any of the studied groups, except for female control mice, which showed age-dependent decline in MCHC values.

### Plasma Lipid Profile of Murine Blood

Both female and male ApoE/LDLR^−/−^ mice showed greatly elevated cholesterol levels (Supplementary Figure SM1A) in comparison to age-matched control mice. Moreover, cholesterol levels were significantly higher in male control mice compared with age-matched females but were significantly lower in male ApoE/LDLR^−/−^ mice in comparison to age-matched females. Control mice showed no age-related differences in cholesterol levels; however, it was elevated with age progression in ApoE/LDLR^−/−^ mice in both female and male groups.

HDL plasma levels (Supplementary Figure SM1B) declined in ApoE/LDLR^−/−^ mice compared with age-matched controls in all studied groups. Male mice exhibited higher HDL levels than age-matched females, except for 24-week-old control mice, which showed no significant difference. Aged mice showed significantly lower HDL levels in all studied groups, except for female control mice, in which no significant difference was observed.

We observed significantly higher LDL levels (Supplementary Figure SM1C) in both female and male ApoE/LDLR^−/−^ mice than in age-matched controls. LDL levels declined in male ApoE/LDLR^−/−^ mice when compared with age-matched females. However, no sex-related difference was found in control mice of both ages. ApoE/LDLR^−/−^ mice showed higher LDL levels with senescence, but no age-related difference was observed in control mice of all studied groups.

Triglycerides levels (Supplementary Figure SM1D) were significantly higher in female and male ApoE/LDLR^−/−^ mice than in their age-matched controls. No significant sex-related difference was observed in any of the studied groups, except for 8-week-old ApoE/LDLR^−/−^ males, which showed higher triglycerides levels when compared with females. No age-related difference was observed in any of the studied groups.

### Basal Intracellular and Extracellular ATP Levels of Murine RBCs

**Figure 3:**
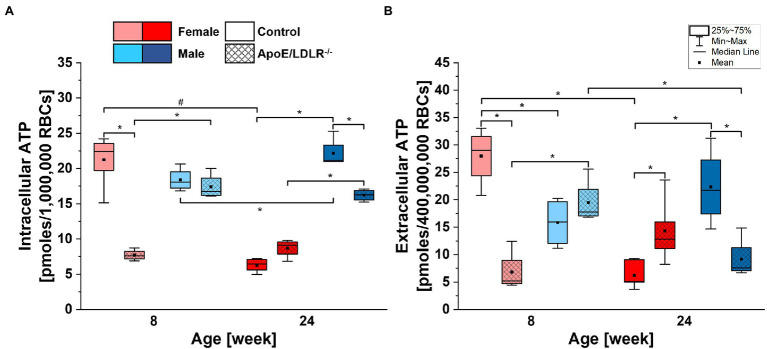
Comparison of basal intracellular **(A)** and extracellular **(B)** adenosine triphosphate (ATP) levels of RBCs isolated from 8- and 24-week-old, female and male, control and ApoE/LDLR^−/−^ mice (*n* = 4–6). Normality was assessed with Shapiro–Wilk test. The data are expressed as box plots (mean, median, and interquartile range with min–max whiskers) and the significance was calculated using Kruskal–Wallis ANOVA test (^*^p < 0.05, ^#^*p* < 0.01). Values were normalized to picomoles per 10^6^ RBCs for basal intracellular ATP levels and to picomoles per 4 × 10^8^ RBCs for basal extracellular ATP levels.

Basal intracellular ATP levels of RBCs (Figure 3A) isolated from ApoE/LDLR^−/−^ mice were significantly declined in 8-week-old female and 24-week-old male mice, while no significant difference was observed in 24-week-old female and 8-week-old male mice, compared to their age-matched controls. We observed significantly higher basal intracellular ATP levels in RBCs isolated from male mice compared with their age-matched female counterparts, except for 8-week-old control mice. Basal intracellular ATP levels of RBCs isolated from control mice significantly declined with age in females while elevated in males. However, no significant age-related difference in basal intracellular ATP levels of RBCs isolated from ApoE/LDLR^−/−^ mice was observed.

Basal extracellular ATP levels of RBCs (Figure 3B) were found to be 400 times lower than corresponding intracellular levels and mirrored basal intracellular ATP levels of RBCs in all studied groups. Therefore, we can state that disease-, sex-, and age-related alterations in basal intracellular ATP levels of RBCs will also translate to basal extracellular ATP levels of RBCs.

**Figure 4:**
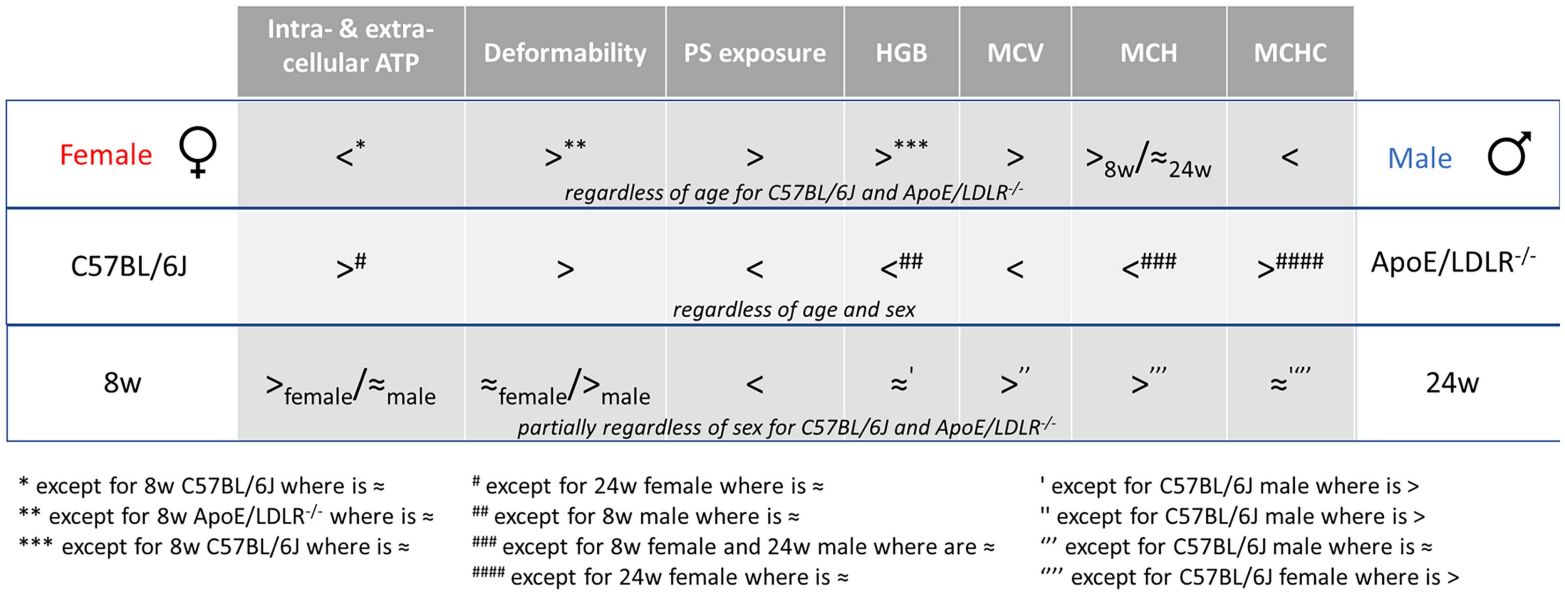
Schematic summary of the sex-, disease-, and age-related differences in RBC parameters. “8w” and “24w” represent the age of mice as 8-week-old and 24-week-old, respectively.

Schematic summary of all our studies concerning the sex-, disease-, and age-related differences in RBC parameters was presented in Figure 4.

## Discussion

Basal intra- and extracellular ATP levels of RBCs acquired from ApoE/LDLR^−/−^ and C57BL/6J mice were evaluated and correlated with sex-related alterations of RBCs in hypercholesterolemia and atherosclerosis. Such comprehensive study was conducted for the first time and proved that intracellular ATP levels differ in females and males RBCs and are sufficient to maintain their viability and efficiency of their crucial functions. Moreover, sex-related differences in basal intracellular ATP levels were correlated partially with changes in RBC morphology and PS exposure on RBC surface (Figure 4).

Sex-related alterations of RBCs are widely evaluated in humans and could be an important risk factor for cardiovascular disease, as well as a promoter for further pathophysiological processes (Kameneva et al., 1999, 2003; Zeltser et al., 2004). Moreover, sex-related differences in RBC parameters were reported in many studies conducted in laboratory animals (Nemeth et al., 2010; Kanias et al., 2011; Guo et al., 2015). Altogether, present observations highlight the significance of the sex of laboratory animals, what should be one of the prominent variables while investigating RBC parameters.

### Mechanical Properties of RBCs

The unique structure of RBC membrane determines size, shape, and mechanical properties of RBCs (Smith, 1987). Approximately 50% of the normal RBC membrane consists of lipids (Buchwald et al., 2000), and it is widely known that mature RBCs are unable to synthesize them, therefore, some of lipid-related membrane changes result from lipid exchange with plasma (Harris et al., 1981). Elevated levels of cholesterol, LDL, and triglycerides alongside declined HDL levels found in plasma of ApoE/LDLR^−/−^ mice when compared to age-matched controls were confirmed (Supplementary Figure SM1), what stays in agreement with our previous findings (Dybas et al., 2020). Similarly, total cholesterol level in plasma was elevated in ApoE/LDLR^−/−^ mice (Csányi et al., 2012), what, together with previously demonstrated lipid profile changes in RBC membrane, proves elevated cholesterol content in RBC membrane of ApoE/LDLR^−/−^ mice compared with age-matched C57BL/6J mice (Dybas et al., 2020). Cholesterol-enriched RBC membrane becomes more rigid and loses its deformability, a crucial feature responsible for maintaining efficient oxygen transport to the cells through microcapillaries (Huisjes et al., 2018). Herein, consistently with previous findings (Kanakaraj and Singh, 1989; Unruh et al., 2015; Dybas et al., 2020), we confirmed deformability loss in RBCs isolated from ApoE/LDLR^−/−^ mice compared to age-matched control, presumably related to the high cholesterol levels in plasma.

Previous studies in both human and laboratory animals reported that RBC parameters vary in a sex-dependent manner (Nemeth et al., 2010; Kanias et al., 2011; Grau et al., 2018; Szczesny-Malysiak et al., 2021). We confirmed that female control mice have higher deformability than age-matched males. Moreover, 24-week-old male ApoE/LDLR^−/−^ mice show lower deformability than 24-week-old female ApoE/LDLR^−/−^ mice, contrary to our previous results that indicated no sex-related difference in RBC deformability in 24-week-old mice (Dybas et al., 2020). RBCs isolated from female mice exhibited higher resistance to oxidative stress (Kanias et al., 2011), for example, long-term hypercholesterolemia exposure (Kampschulte et al., 2014), which may imply higher membrane integrity of RBCs in females than in males. Such difference could be a result of the protective effect of female sex hormones (Kanias et al., 2016; Grau et al., 2018), and/or the effect of testosterone in male mice that could increase susceptibility to hemolysis (Kanias et al., 2011, 2016), that is, promote loss of RBC membrane integrity. However, we did not observe any significant sex-related difference in 8-week-old ApoE/LDLR^−/−^ mice that may indicate the protective effect of female sex hormones is less effective than the effect of higher plasma cholesterol level in females, which, in turn could increase RBC membrane rigidity (Buchwald et al., 2000).

Additionally, we confirmed the loss of deformability in males progressing with age, what is in line with our previous study (Dybas et al., 2020). However, no loss of RBC deformability in females was observed, what could be correlated with their higher resistance during exhibition to oxidative stress (Kanias et al., 2011).

### Alterations of RBC Membrane Asymmetry

Red blood cell membrane exhibits an asymmetric distribution of phospholipids (Schroit and Zwaal, 1991). Maintaining this asymmetry is critically important for survival of RBCs, because the loss of phospholipid asymmetry, especially the appearance of PS on the outer leaflet of RBC membrane, is a signal for eryptosis (Segawa and Nagata, 2015). Lipid exchange between plasma and RBC membrane has a major role in PS exposure (Van Zwieten et al., 2012). RBCs incubated in cholesterol-enriched environment results in higher PS exposure (López-Revuelta et al., 2007; Harisa and Badran, 2015; Unruh et al., 2015). In the present study, we confirmed that RBCs isolated from ApoE/LDLR^−/−^ mice showed higher PS exposure levels on their outer membrane in all studied groups, what corresponded to changes in cholesterol levels.

Our results indicate that PS exposure on RBC membrane was higher in female mice when compared to age-matched males in all studied groups, contrary to previous studies in human RBCs (Straface et al., 2011; Szczesny-Malysiak et al., 2021), where PS exposure on the RBC surface was higher in men. On the other hand, it has been shown that *in vitro* addition of sex hormones has no significant effect on PS exposure levels in human RBCs. (Kanias et al., 2016) Higher PS exposure in female ApoE/LDLR^−/−^ mice could be explained by the reported effect of hypercholesterolemia. (López-Revuelta et al., 2007; Unruh et al., 2015) However, female control mice showed lower plasma cholesterol levels and higher PS exposure in comparison to age-matched males.

### Morphological Changes of RBCs

#### Hemoglobin Concentration

Higher HGB values reported for ApoE/LDLR^−/−^ mice could be a result of the elevated number of circulating RBCs (Supplementary Table SM1). However, MCH values were also elevated in ApoE/LDLR^−/−^ mice, what indicates that RBCs isolated from ApoE/LDLR^−/−^ mice contain more hemoglobin, namely, are hyperchromic. Normally, higher hemoglobin content helps to elevate tissue oxygenation under various circumstances that could deteriorate oxygen transport. In atherosclerosis, thickened arterial walls may cause impaired transport of oxygen. Moreover, hypercholesterolemia increases cholesterol content of RBC membrane, what impacts its rigidity and hinder oxygen diffusion through membrane, thus impairing oxygen transport (Buchwald et al., 2000). Altogether, it may be concluded that higher hemoglobin content in RBCs isolated from both, female and male ApoE/LDLR^−/−^ mice, could be a result of atherosclerosis progression, as a reaction toward an increase in tissue oxygenation.

Red blood cells in men contain more hemoglobin than in women (Murphy, 2014; Grau et al., 2018), what is associated with higher testosterone level that facilitates erythropoiesis (Coviello et al., 2008). The effect of testosterone on hemoglobin content reported for murine RBCs is similar (Guo et al., 2013). In contrast to studies in humans, yet in agreement with previous studies in mice (Leuenberger and Kunstyr, 1976; Raabe et al., 2011; Jackson Laboratory, 2016), our results indicate that HGB values in RBCs were significantly higher in female mice in all studied groups, except for 8-week-old control mice, which showed no significant sex-related difference. The effect of testosterone may explain lower HGB value demonstrated for male ApoE/LDLR^−/−^ mice and no significant difference in HGB value in 8-week-old control mice, since low level of testosterone was reported in those groups (Eleftheriou and Lucas, 1974; Steinfeld et al., 2018). Yet, it is not enough to explain the results obtained in 24-week-old control male mice. Higher hemoglobin content in female ApoE/LDLR^−/−^ mice when compared to males could also be associated with an effect of hypercholesterolemia (Buchwald et al., 2000), since female ApoE/LDLR^−/−^ mice exhibited higher plasma cholesterol levels.

Hemoglobin concentration values decreased with age in all studied groups, but statistical significance was previously documented only in male control mice (Guo et al., 2014). The same study also reported that incorporation of iron into RBCs was significantly lower in older mice, what may explain the decline in HGB values with age.

#### MCV, MCH, and MCHC

The relationship between serum cholesterol concentration and MCV varies in different studies. It has been shown that hypercholesterolemia elevated MCV values in human subjects (Prasad, 2010), although other studies indicated lack of any effect of hypercholesterolemia (Choi and Pai, 2004) or even an inverse correlation (Bunyaratvej et al., 2000). Additionally, a recent study in rabbits indicated that MCV value of RBCs was significantly elevated in hypercholesterolemia (Tanczos et al., 2021). MCV values were significantly elevated in ApoE/LDLR^−/−^ mice, what stands in line with different studies in murine models (Holm et al., 2002; Hoekstra et al., 2013), as well as with our previous work (Dybas et al., 2020). MCV was associated with endothelial function in a previous study in human subjects; therefore, elevated MCV values may reflect the severity of atherosclerosis (Solak et al., 2013). However, contrary to this conclusion, our findings indicate that MCV values declined with age in both male and female ApoE/LDLR^−/−^ mice, despite the severity of atherosclerosis increased (Bar et al., 2019).

Alterations in MCH values mirrored changes in MCV values in all studied groups, as shown previously (Kauffmann and Evans, 2021). Namely, MCH values were augmented in ApoE/LDLR^−/−^ mice as a result of hypercholesterolemia (Prasad, 2010).

Mean corpuscular hemoglobin concentration value, which is related to the hydration level (Gallagher, 2017) and/or internal viscosity (Williams and Morris, 1980) of RBC, was declined in ApoE/LDLR^−/−^ mice compared to age-matched controls, what indicates lower RBC hydration, as an expected result of hypercholesterolemia (Prasad, 2010), except for 24-week-old female mice, where no significant change was observed.

Contrary to our results, previous studies reported that red cell indices, that is, MCV, MCH, and MCHC, showed no sex-related difference in control males (Raabe et al., 2011; Jackson Laboratory, 2016). However, different studies reported red cell indices values for ApoE/LDLR^−/−^ mice, separately for females (Bar et al., 2019) and males (Dybas et al., 2020), that stand in line with our findings. We observed significantly higher MCV values and significantly lower MCHC values in RBCs isolated from female mice in all studied groups. MCH values were significantly higher in 8-week-old female mice, but no difference was observed in case of 24-week-old mice.

We confirmed an age-dependent significant decline in MCV values accompanying MCH values in RBCs isolated from both females and males, what stands in line with our previous study (Dybas et al., 2020) except from male control mice where no significant change was observed. Additionally, no significant age-related differences were observed in MCHC level of RBCs, except from the female control mice, where we observed lowering of the MHCH with age of animals.

### Correlation Between Intracellular ATP Levels and Alterations of Murine RBCs

Adenosine triphosphate is essential for RBCs to maintain their viability, as well as to preserve shape of the cell, to maintain deformability, and to stabilize phospholipid asymmetry of RBC membrane (Feo and Mohandas, 1977; Seigneuret and Devaux, 1984; Betz et al., 2009; Park et al., 2010; Xu et al., 2019). On the one hand, it was previously documented that intracellular ATP levels in human were not sex-dependent (Racine and Dinenno, 2019). On the other hand, there are reports indicating that sex hormones influence intracellular ATP levels in human RBCs (Solomon and Hendler, 1987; Alexander et al., 2021). Our findings show that male mice have higher intracellular ATP levels in RBCs compared with female mice (Figure 3A), except for 8-week-old control mice. No significant age-related difference in basal intracellular levels was observed, neither in female nor in male ApoE/LDLR^−/−^ mice, contrary to C57BL/6J mice, which showed significant variation between females and males, progressing with age. The basal intracellular ATP levels of RBCs isolated from 8-week-old ApoE/LDLR^−/−^ mice showed a decrease compared to age-matched controls that was more prominent in females than in males. Such findings for 8-week-old ApoE/LDLR^−/−^ mice could be related to the higher plasma cholesterol levels in females. Similar differences in cholesterol levels observed also in 24-week-old ApoE/LDLR^−/−^ mice where atherosclerosis disease developed, however, the basal intracellular ATP levels of RBCs, were lower compared to age-matched controls only in males. Although the level of plasma cholesterol could affect the intracellular ATP levels of RBCs, above converse findings may suggest that no direct correlation consist between the plasma cholesterol levels and the basal intracellular ATP levels of RBCs in ApoE/LDLR^−/−^ mice. However, further studies could be realized to dissolve this relation and may uncover novel mechanisms.

As presented in Figure 3, basal intracellular ATP levels in RBCs showed a narrower distribution than basal extracellular ATP levels. However, it must be noted that the determined intracellular ATP levels were expressed per 1,000,000 RBCs, while extracellular ATP levels were expressed per 400,000,000 RBCs. Basal extracellular ATP level correlates strongly with basal intracellular ATP level in healthy human RBCs (Kirby et al., 2014). Herein, we demonstrated a similar correlation between basal intracellular and extracellular ATP levels in murine RBCs that mirrored observed tendencies. The presence of this relationship appears to be independent of disease, sex, and age of mice. Accordingly, one can conclude that basal intracellular ATP level in RBCs is one of the major variables that affects basal extracellular ATP levels of RBCs in murine models.

Although deformability and intracellular ATP level are both sex-related, their correlation is unfortunately inconclusive. In this study, intracellular ATP levels increased with age in male control mice, while male ApoE/LDLR^−/−^ mice showed no significant difference. However, RBC deformability significantly declined in aged male mice. In females, intracellular ATP levels greatly declined with age in control group but did not change in ApoE/LDLR^−/−^ mice. RBC deformability showed no age-dependent difference in females nor in control neither in ApoE/LDLR^−/−^ mice. In general, intracellular ATP levels were higher, but RBC deformability was lower in males when compared to age-matched females. Collectively, we may conclude that there is no noticeable link between intracellular ATP level and deformability of RBCs, which has been reported previously (Karger et al., 2012). However, this correlation was reviewed in detail and its presence has been assigned to the method of deformability measurements (Huisjes et al., 2018). Therefore, further investigations using different methods to assess RBC deformability would be beneficial to display this correlation conclusively.

Numerous studies show that PS exposure on RBC membrane was strongly correlated with intracellular ATP level (Seigneuret and Devaux, 1984; Boas et al., 1998; Nagata et al., 2016). We demonstrated a similar trend. In general, female and ApoE/LDLR^−/−^ mice showed significantly higher PS exposure, but lower intracellular ATP levels, compared to age-matched males and control mice, respectively.

The effect of intracellular ATP level on the morphological aspects in RBCs was also reported (Feo and Mohandas, 1977; Park et al., 2010). The most explicit parameter was MCV that indicates the mean volume of an RBC. Lowered ATP levels could cause a decline in the activity of Na^+^/K^+^-ATPase channel, what may result in ion imbalance, for example, elevated level of intracellular Na^+^, and therefore cause volume alterations in RBCs (Gadsby et al., 2012). Such mechanism may correlate the displayed intracellular ATP levels and MCV values in females and ApoE/LDLR^−/−^ mice.

In conclusion, intracellular ATP levels of RBCs correspond with the trends of changes observed for extracellular ATP levels, MCV, and PS exposure, but differ from trends observed for HGB, MCH, MCHC, and deformability. The changes were sex-dependent and therefore it would be beneficial for further studies to consider sex of laboratory animals as a variable of RBC physiology.

### Study Limitation Section

Our study used C57BL/6J as a control which is recommended as one of the appropriate controls by The Jackson Laboratory next to other possible strain of mice—129/SvJ. It is known that ApoE/LDLR^−/−^ mice are derived from C57BL/6J × 129/SvJ strain, hence applicability of both these strain as a control is justified. However, it would be beneficial to compare results to both these control mice strains in our further studies. The limitation of the application of ApoE/LDLR^−/−^ mice as a model of human condition is that although atherosclerotic lesions occur, these lesions rarely progress to the advanced stages of atherothrombotic vascular occlusion in mice as observed in humans.

## Data Availability Statement

The datasets presented in this study can be found in online repositories. The names of the repository/repositories and accession number(s) can be found at: https://doi.org/10.6084/m9.figshare.17008216.v1.

## Ethics Statement

All animal procedures were performed in accordance with the Guide for the Care and Use of Laboratory Animals published by the United States National Institutes of Health (NIH Publication No. 85–23, revised 1985) as well as with the guidelines of the Local Ethical Committee on Animal Experiments in Krakow. Because only mice blood was used for the experiments (no additional procedure was performed besides sacrifice and blood collection), no additional number from the Local Ethical Committee on Animal Experiments in Krakow was necessary.

## Author Contributions

FA: conceptualization, methodology, validation, investigation, formal analysis, visualization, data curation, and writing—original draft. TM: methodology, investigation, formal analysis, visualization, and writing—review and editing. KB: investigation, formal analysis, visualization, and writing—review and editing. JD and ES-M: investigation, formal analysis, and writing—review and editing. MK: investigation. MF-Z and RK: resources. KM: conceptualization, methodology, writing—review and editing, project administration, supervision, and funding acquisition. All authors contributed to the article and approved the submitted version.

## Funding

This research was funded by the Polish National Science Centre, the OPUS grant No. UMO 2016/23/B/ST4/00795.

## Conflict of Interest

The authors declare that the research was conducted in the absence of any commercial or financial relationships that could be construed as a potential conflict of interest.

## Publisher’s Note

All claims expressed in this article are solely those of the authors and do not necessarily represent those of their affiliated organizations, or those of the publisher, the editors and the reviewers. Any product that may be evaluated in this article, or claim that may be made by its manufacturer, is not guaranteed or endorsed by the publisher.
