# Supplementary material for: Sex-Specific Differences of Adenosine Triphosphate Levels in Red Blood Cells Isolated From ApoE/LDLR Double-Deficient Mice
Source: Front Physiol. 2022 Feb 18;13:839323. doi: 10.3389/fphys.2022.839323 (PMC8895041; doi:10.3389/fphys.2022.839323)
Supplement: Supplementary file 1 [file Data_Sheet_1.docx]

Supplementary Material

**Sex-specific differences of adenosine triphosphate (ATP) levels in red blood cells isolated from ApoE/LDLR double-deficient mice**

Fatih Celal Alcicek^1^, Tasnim Mohaissen^1,2^, Katarzyna Bulat^1,3^, Jakub Dybas^1^, Ewa Szczesny-Malysiak^1^, Magdalena Kaczmarska^1^, Magdalena Franczyk-Zarow^4^, Renata Kostogrys^4^, Katarzyna M. Marzec^1,3*^

^1^Jagiellonian Centre for Experimental Therapeutics, Jagiellonian University, Krakow, Poland

^2^Chair and Faculty of Pharmacy, Jagiellonian University Medical College, Krakow, Poland

^3^Łukasiewicz Research Network - Krakow Institute of Technology, Krakow, Poland

^4^Department of Human Nutrition and Dietetics, Faculty of Food Technology, University of Agriculture, Krakow, Poland

|  | Female | | | | Male | | | |
| --- | --- | --- | --- | --- | --- | --- | --- | --- |
|  | C57BL/6J | | ApoE/LDLR^-/-^ | | C57BL/6J | | ApoE/LDLR^-/-^ | |
|  | 8-week-old | 24-week-old | 8-week-old | 24-week-old | 8-week-old | 24-week-old | 8-week-old | 24-week-old |
| RBC [mln mm^-3^] | 9.37 ± 0.30 | 10.18 ± 0.65 | 10.30 ± 0.06 | 10.92 ± 0.88 | 9.95 ± 0.49 | 8.27 ± 0.86 | 9.69 ± 0.67 | 9.53 ± 0.66 |
| HGB [g dL^-1^] | 14.85 ± 0.42 | 14.44 ± 0.70 | 16.51 ± 0.11 | 16.27 ± 1.06 | 14.35 ± 0.72 | 12.20 ± 0.36 | 15.06 ± 1.08 | 14.03 ± 1.02 |
| HCT [%] | 55.47 ± 5.74 | 54.86 ± 5.22 | 63.65 ± 2.82 | 59.63 ± 4.42 | 48.39 ± 2.39 | 40.86 ± 1.70 | 51.76 ± 3.56 | 48.43 ± 3.60 |
| MCV [um^3^] | 55.3 ± 1.49 | 50.57 ± 1.29 | 61.43 ± 2.06 | 54.71 ± 1.29 | 48.88 ± 0.78 | 48.00 ± 2.00 | 53.33 ± 1.15 | 50.88 ± 0.78 |
| MCH [pg] | 15.85 ± 0.26 | 14.20 ± 0.37 | 16.03 ± 0.35 | 14.92 ± 0.38 | 14.45 ± 0.19 | 14.36 ± 0.55 | 15.45 ± 0.11 | 14.69 ± 0.25 |
| MCHC [g dL^-1^] | 29.02 ± 0.30 | 26.48 ± 1.84 | 26.18 ± 0.74 | 27.32 ± 1.13 | 29.65 ± 0.30 | 30.04 ± 0.51 | 29.09 ± 0.43 | 28.96 ± 0.30 |
| RDW [%] | 10.76 ± 0.21 | 11.10 ± 0.25 | 10.61 ± 0.21 | 10.30 ± 0.15 | 14.44 ± 0.59* | 14.05 ± 0.34 | 13.29 ± 0.30* | 12.51 ± 0.50 |

**Supplementary Table 1.** Complete blood count measurements of whole blood samples withdrawn from 8- and 24-weeks-old, female and male, C57BL/6J and ApoE/LDLR^-/-^ mice (N=5-9). The RDW data marked with * are related to 5-weeks-old mice instead of 8-weeks-old. The data presented as Mean ± SD.


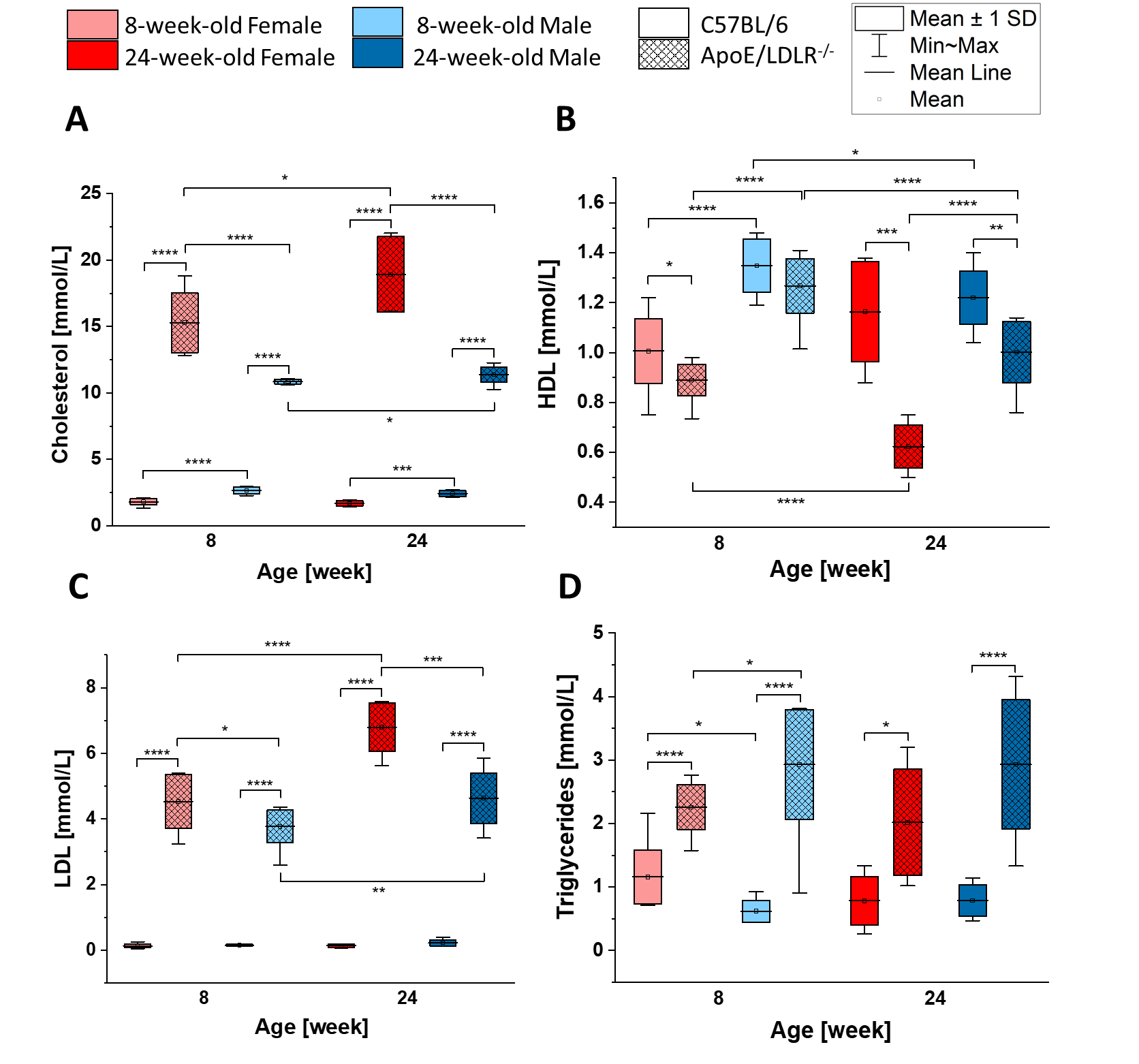


**Fig. SM 1**. Cholesterol (A), HDL (B), LDL (C) and Triglycerides (D) level in blood plasma from 8– and 24–weeks‒old, female and male, C57BL/6J and ApoE/LDLR^‒/‒^ mice. Normality was assessed using Shapiro‐Wilk test. The data are expressed as box plots (mean, SD, and min-max whiskers) and the significance was calculated with Mann–Whitney test (*p<0.05, **p<0.01, ***p<0.001, ****p<0.0001).
